# Bridging the Gap: Medical Schools’ Transition to Residency Courses and their Graduates’ Readiness for Residency

**DOI:** 10.1007/s40670-026-02787-4

**Published:** 2026-07-07

**Authors:** Lauren A. Heidemann, Douglas Grbic, Dorothy Andriole, Matthew Rustici, Kathryn Mutter, Genie Roosevelt, Heather Davis, Jason C. Brainard

**Affiliations:** 1 University of Michigan Medical School, Ann Arbor, MI USA; 2 Medical Education Research, Association of American Medical Colleges, Washington, DC USA; 3 University of Colorado School of Medicine, Aurora, CO USA; 4 University of Virginia School of Medicine, Charlottesville, VA USA; 5 David Geffen School of Medicine at UCLA, Los Angeles, CA USA; 6 Department of Internal Medicine, UH South Unit 4, 1500 E Medical Center Dr, Ann Arbor, MI 48109-5220 USA

**Keywords:** Transition to residency, Resident readiness, Medical school curriculum, Undergraduate medical education

## Abstract

Transition To Residency (TTR) courses prepare U.S. medical school graduates for residency. Program director assessments of post-graduate year-1 (PGY-1s) readiness for residency (“did not meet [DNM]” vs. “met/exceeded” expectations) were stratified by medical school TTR course presence. Of 9,214 PGY-1s graduating in 2023–2024 from medical schools that completed the TTR Educators survey and participated in the Association of American Medical College Resident Readiness Survey, 2.4% (221/9,214) DNM expectations. Adjusting for specialty, PGY-1s from schools with (vs. without) TTR courses had lower odds of “DNM” expectations (odds ratio 0.62; 95% confidence interval 0.40–0.97). TTR courses may support resident readiness across specialties.

## Background

Transition To Residency (TTR) courses, implemented at many U.S. medical schools [1], intend to bridge the gap between undergraduate medical education (UME) and the start of post-graduate-year (PGY)-1 training [2]. TTR courses aim to help students consolidate prior knowledge gained in medical school and further develop advanced skills to prepare for residency. Among those U.S. medical schools that offer TTR courses, TTR course content varies by medical school and typically includes topics such as responding to common inpatient issues and pages from bedside nurses, responding to rapid responses and codes, communicating patient handoffs, and procedural training [1]. Approximately two-thirds of schools that offer TTR courses provide distinct specialty-specific training for graduating students, with the remaining schools offering a single course for all students regardless of intended specialty [1]. Beyond variations in specialty-specific training, TTR courses can also vary in duration, assessment structure, and requirement status; in a recent national survey by Heidemann et al., 79.1% of TTR courses were required [1].

Previous studies on TTR course effectiveness, mainly focusing on student knowledge acquisition and self-perceived residency preparedness, have yielded mixed results [2–5]. In this retrospective cross-sectional study, we sought to determine if Program Director (PD) assessments of their PGY-1 trainees’ (“PGY-1s”) readiness for residency, as reported on the Association of American Medical College (AAMC) Resident Readiness Survey (RRS) [6], were associated with TTR course offerings at the medical schools the PGY-1s had attended.

## Activity

Our study-eligible population included academic year (AY) 2024–2025 PGY-1s who graduated in AY 2023–2024 from U.S. MD-granting schools that responded to the 2024 TTR Educators survey [1] item about TTR course offerings (118/151 invited schools; 78% response rate), and also chose to participate in the 2024–2025 AAMC RRS process (118/118 invited schools; 100%). Using TTR Educators survey data, we created a dichotomous variable (yes vs. no) for whether the medical school offered any TTR course(s). Based on RRS data, we created a 7-category co-variable for graduate medical education (GME) program specialty category (as PD assessments of resident readiness vary by specialty [7]) and a dichotomous variable for our outcome of interest: PD response to the RRS item: “During the transition to GME (0–6 months of PGY-1 year), did this resident meet overall performance expectations?” (“did not meet” vs. “met/exceeded”). We used Chi-square tests to assess bivariate associations and multivariable logistic regression to identify independent associations between each variable and the outcome, reporting adjusted odds ratios (aOR) and 95% Confidence Intervals (CI). The Human Subjects Offices of the AAMC and the University of Michigan exempted the study from institutional board review.

## Results

There were 14,776 study- eligible PGY-1s from 118 schools. Of these 118 schools, 110 schools (110/118, 93.2%) offered TTR courses and 8 schools (8/118, 6.8%) did not offer TTR courses. Of the 14,776 study-eligible PGY-1s, there were 9,214 PGY-1s (9,214/14,776, 62.4%) with RRS data for our outcome of interest comprised our study sample. These 9,214 PGY-1s included 8,626 (93.6%) PGY-1s from the 110 schools that offered TTR courses, and 588 (6.4%) PGY-1s from the 8 schools that did not. Overall, 2.4% (221/9,214) of all PGY-1s in our study sample did not meet PD expectations (Table 1). As shown, this proportion varied by medical-school TTR course status: 3.7% (22/588) of PGY-1s from schools without TTR courses, and 2.3% (199/8626) of PGY-1s from schools with TTR courses did not meet PD expectations (*P* = 0.028). The proportion of PGY-1s in our study sample who did not meet overall PD expectations also varied by specialty category (*P* < 0.001), ranging from 1.3% (28/2232) of PGY-1s in the “Other non-surgical specialties” category to 4.4% (33/747) of PGY-1s in the “General Surgery” category. Not shown in Table 1, among the 8,626 PGY-1s from schools with TTR courses, this outcome did not vary by course duration (1–3 weeks, 2.3% [133/5,685]); ≥4 weeks, 2.2% [63/2,841], duration not specified, 3.0% [3/100]; *P* = 0.843).

**Table 1:** Characteristics of study sample grouped by outcome

	Total sample, No.(%)(*N* = 9214)^a^	Did not meet PD expectations, No.(%)(*n* = 221) ^b^	Met/exceeded PD expectations, No.(%)(*n* = 8993) ^b^	*P* value
TTR course(s) offered at medical school attended				0.028
Yes	8626 (93.6)	199 (2.3)	8427 (97.7)	
No	588 (6.4)	22 (3.7)	566 (96.2)	
GME program specialty category				< 0.001
Internal medicine^c^	712 (7.7)	17 (2.4)	695 (97.6)	
Emergency medicine	954 (10.4)	36 (3.8)	918 (96.2)	
Family medicine	2225 (24.1)	58 (2.6)	2167 (97.4)	
Obstetrics and gynecology	594 (6.4)	18 (3.0)	576 (97.0)	
Pediatrics	817 (8.9)	18 (2.2)	799 (97.8)	
General surgery	747 (8.1)	33 (4.4)	714 (95.6)	
Other surgical specialties^d^	933 (10.1)	13 (1.4)	920 (98.6)	
Other non-surgical specialties^e^	2232 (24.2)	28 (1.3)	2204 (98.7)	

Abbreviations: *TTR* transition to residency *GME* graduate medical education

^a^ Percentages shown are within-variable column percentages. Percentages may not add up to 100 due to rounding

^b^ Percentages shown are within-variable row percentages. Percentages may not add up to 100 due to rounding

^c^ Includes both Internal medicine and Internal Medicine-Pediatrics (combined program)

^d^ Other surgical specialties included Neurological surgery, Orthopedic surgery, Otolaryngology, Plastic surgery, Thoracic surgery, Urology, and Vascular surgery

^e^ All other nonsurgical specialties (e.g., Dermatology, Neurology, Pathology–anatomic and clinical, and Psychiatry)

As shown in Table 2, PGY-1s from schools with (vs. without) TTR courses had *lower* odds of not meeting expectations (vs. meeting/exceeding) (aOR 0.62; 95% CI, 0.40–0.97; *P* = 0.037). On a specialty category basis (each vs. Internal Medicine/Medicine-Pediatrics reference group), odds of not meeting expectations were *higher* for PGY-1s in General Surgery (aOR 1.72; 95% CI, 1.11–2.66, *P* = 0.014).Odds of not meeting expectations were *lower* for PGY-1s in Other non-surgical specialties (aOR 0.48; 95% CI, 0.30–0.75, *P* = 0.001) and Other surgical specialties (aOR 0.53; 95% CI, 0.29–0.98, *P* = 0.042). Odds were *similar* (each *P* > 0.05) for PGY-1s in each of Emergency medicine, Family medicine, Obstetrics & Gynecology, and Pediatrics.

**Table 2:** Logistic regression result for 221 PGY-1 residents who did not meet vs. 8993 PGY-1 residents who met or exceeded expectations of 9214 total PGY-1 residents^a^

	AOR (95% CI)	*P* value
TTR course(s) offered		
Yes	0.62 (0.40–0.97)	0.037
No	1 [Reference]	
GME program specialty category		
Internal medicine ^a^	1 [Reference]	
Emergency medicine	0.92 (0.53–1.60)	0.773
Family medicine	1.47 (0.96–2.24)	0.076
Obstetrics and gynecology	1.17 (0.69–2.01)	0.561
Pediatrics	0.85 (0.50–1.45)	0.555
General surgery	1.72 (1.11–2.66)	0.014
Other surgical specialties^b^	0.53 (0.29–0.98)	0.042
Other non-surgical specialties^c^	0.48 (0.30–0.75)	0.001

Abbreviations: *AOR* adjusted odds ratio, *CI* confidence interval, *TTR* transition to residency, *GME* graduate medical education

^a^ Includes both Internal medicine and Internal Medicine-Pediatrics (combined program)

^b^ Other surgical specialties included Neurological surgery, Orthopedic surgery, Otolaryngology, Plastic surgery, Thoracic surgery, Urology, and Vascular surgery

^c^ Other nonsurgical specialties (e.g., Dermatology, Neurology, Pathology–anatomic and clinical, and Psychiatry)

## Discussion

In this national cross-sectional study, we identified an independent association between graduation from medical schools with TTR courses and PGY-1s’ readiness for the transition to residency, after adjusting for GME program specialty. To our knowledge, this is the first national, multispecialty study to demonstrate a relationship between TTR course offerings at the medical school level and preparedness for residency as reported by PDs (i.e. not self-report). Our observation suggests that TTR courses may provide value in supporting resident readiness across a range of specialties.

The overall prevalence of not meeting expectations was low in our study sample, consistent with others’ observations for different study cohorts [5]. Although the prevalence was low, it is important to note that struggling PGY-1s likely have a profound impact on many aspects of the overall training program. These extend beyond the personal and professional impacts for the individual PGY-1. We speculate that even small shifts in decreasing the likelihood of early underperformance among PGY-1s may be broadly beneficial for residency programs and, importantly, patient care.

Our data shows that trainees entering General Surgery had higher odds of not meeting expectations (compared to those entering IM/IM-Pediatrics reference group). This is consistent with prior observations [7] and may reflect increased responsibilities expected of surgical trainees (including procedural and technical prowess) and/or higher baseline expectations from PDs. Our specialty observations reiterate the role for continuing collaboration between GME educators and UME educators in tailoring specialty-specific approaches to TTR content. For example, multiple collaborative national resources exist for students entering Surgical specialties [8], Obstetrics & Gynecology [9], Emergency Medicine [10], and Other specialties [11]. Further research is warranted to determine if providing specialty-specific course material for graduating medical students reduces the variability seen between specialties in the RRS outcome.

Our study has numerous limitations. Our findings describe correlations only; causality cannot be inferred. The association that we observed for TTR course offerings and PGY-1s’ preparedness for the transition to residency may be due to other aspects of schools that offer TTR courses, and their students, rather than TTR course offerings per se. Additionally, the denotation of “met expectations” is subjective based on the Program Director’s judgment, which may be influenced by specialty and institutional norms. Furthermore, using TTR course presence/absence and duration as binary variables does not account for the significant heterogeneity of these courses. With this in mind, various aspects of medical-school TTR courses (e.g., specialty-specific vs. generic content and/or tracks, required vs. elective, curricular components), in the context of other individual-level and institutional characteristics, warrant investigation to more fully delineate the relationship between medical-school TTR course offerings and their graduates’ preparedness for residency across all specialties. Finally, further study is also warranted to investigate, on a specialty-specific basis, the extent to which specific TTR course types and content, as well as other specialty-specific interventions to optimize the transition to residency, may be associated with PGY-1 preparedness as assessed by their PDs.
